# COVID-19 and the gender health paradox

**DOI:** 10.1177/1403494820975604

**Published:** 2020-12-14

**Authors:** Clare Bambra, Viviana Albani, Paula Franklin

**Affiliations:** Population Health Sciences Institute, Newcastle University, UK

**Keywords:** Coronavirus, social determinants of health, health inequalities, health equity, health disparities, women, men, sex

## Abstract

This article examines gender-based health inequalities arising from the COVID-19 pandemic by drawing on insights from research into the ‘gender health paradox’. Decades of international research shows that, across Europe, men have shorter life expectancies and higher mortality rates than women, and yet, women report higher morbidity. These gender-based health inequalities also appear to be evident within the pandemic and its aftermath. The article starts by providing an overview of the ‘gender health paradox’ and the biological, social, economic and political explanations for it. It then outlines the international estimates of gender-based inequalities in COVID-19 morbidity and mortality rates – where emerging data suggests that women are more likely to be diagnosed with COVID-19 but that men have a higher mortality rate. It then explores the longer term consequences for gender-based health inequalities of the aftermath of the COVID-19 pandemic, focusing on the impacts of government policy responses and the emerging economic crisis, suggesting that this might lead to increased mortality amongst men and increased morbidity amongst women. The essay concludes by reflecting on the pathways shaping gender-based health inequalities in the COVID-19 pandemic and the responses needed to ensure that it does not exacerbate gender-based health inequalities into the future.

## Introduction

From 1 January to 1 June 2020, there have been 1.4 million confirmed cases and 165,000 confirmed deaths from the COVID-19 pandemic in Europe. Emerging estimates suggest that women are more likely to be diagnosed with COVID-19, but men have higher mortality. This article examines these gender differences in experiences of the COVID-19 pandemic by drawing on insights from longstanding, international research into the ‘gender health paradox’[1]. Decades of international research show that, across Europe, men have shorter life expectancies and higher mortality rates than women, and yet, women report higher morbidity – or, to put it more simply, ‘women get sicker, men die quicker’ [2]. This article examines the implications of the COVID-19 pandemic for these gender-based inequalities in health.

The article is divided into three parts. Part one outlines the ‘gender health paradox’ and the biological, social, economic and policy explanations for it. In part two, it summarises contemporary international estimates of gender-based inequalities in COVID-19 morbidity and mortality – where emerging data suggests that women are more likely to be diagnosed with COVID-19 but that men have a higher mortality rate. Part three explores the longer-term consequences for gender-based health inequalities of the COVID-19 pandemic, focusing on the impacts of government policy responses and the emerging economic crisis. The conclusion reflects on the pathways shaping gender-based health inequalities in the COVID-19 pandemic and the responses needed to ensure that it does not exacerbate gender-based health inequalities into the future.

## The gender health paradox

**Figure 1.**
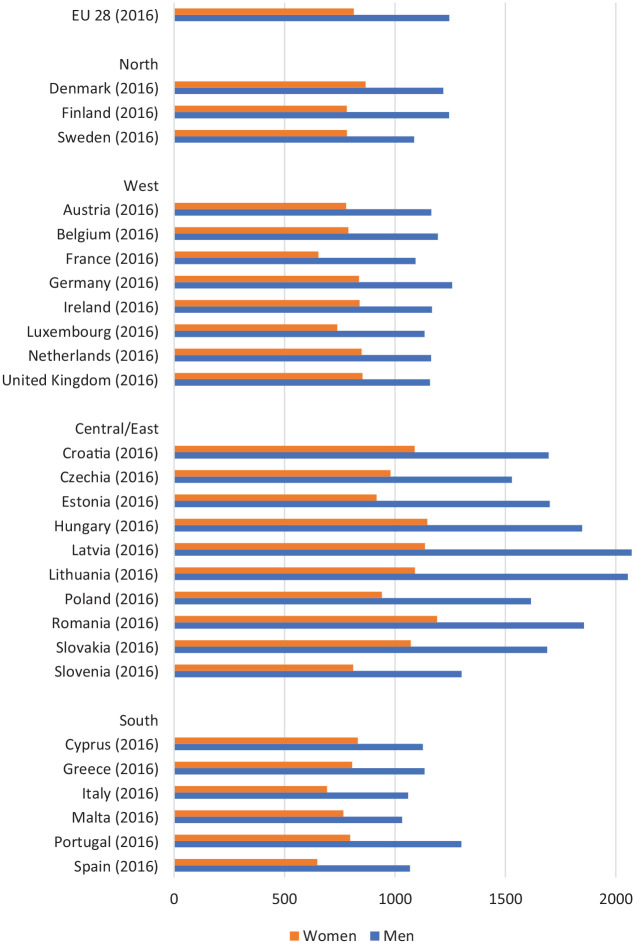
Age-standardised all-cause mortality rates by gender, per 100,000 inhabitants; 28 EU member states (2016). Source: Eurostat (2016) [4].

In Europe, men have lower life expectancies than women, but women spend their extra years with higher levels of ill health: the so-called ‘gender health paradox’ [1]. Men’s mortality disadvantage in Europe is evident across all-cause mortality, life expectancy, cardiovascular disease and cancer mortality, as well as deaths from violence and suicide [3]. For example, as Figure 1 shows, all-cause mortality rates are significantly higher for men in all 28 European Union countries [4]. Men are also more likely to engage in risky health behaviours such as excessive alcohol consumption, drug use and smoking [3]. These gender differences are fairly stable throughout the life course, although, there is emerging evidence which suggests that amongst younger age groups, risky health behaviours are more equal with the gender gap in smoking and alcohol consumption closing [5,6]. Men’s mortality disadvantage is particularly evident in the countries of Central and Eastern Europe – potentially related to excess alcohol consumption [7]. There are also larger gender differences in mortality amongst men and women in the lowest socio-economic groups [8].

**Figure 2.**
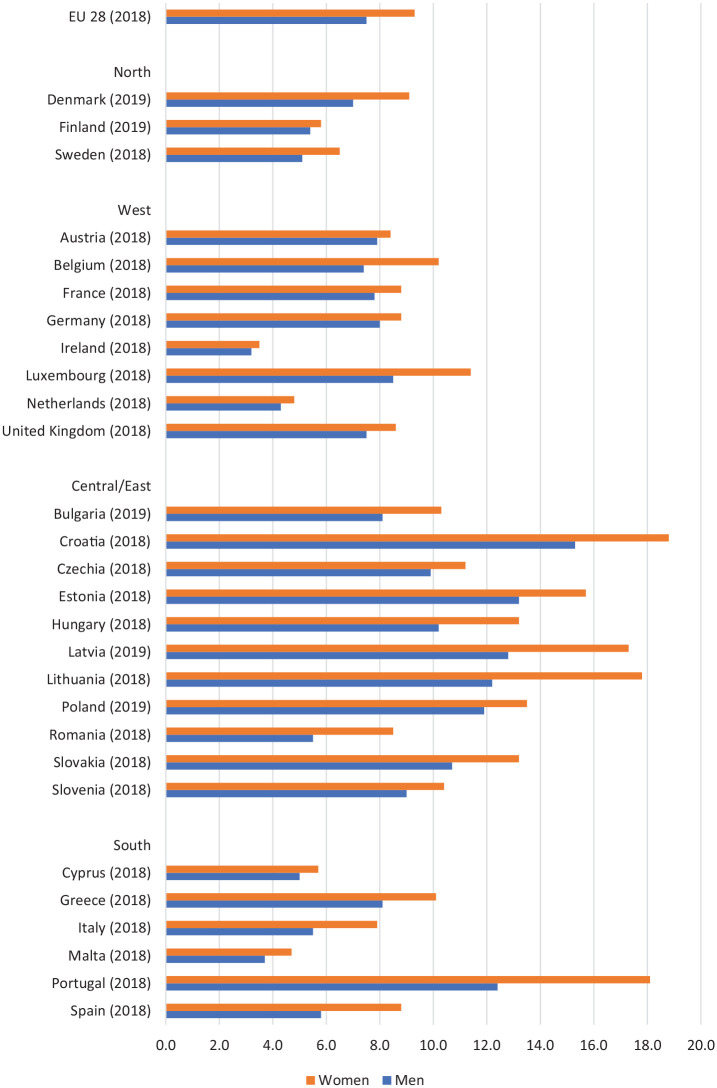
Proportion (%) of men and women aged 16 years and over reporting ‘bad or very bad’ self-perceived health symptoms; 28 EU member countries. Source: Eurostat (2020) [10].

**Figure 3.**
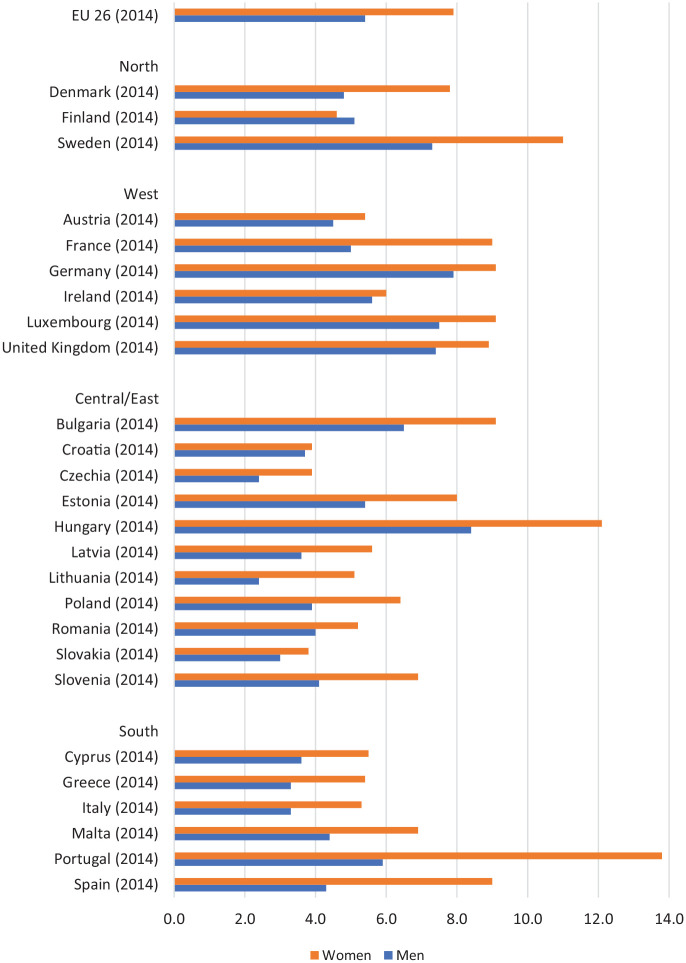
Proportion (%) of men and women aged 15 years and over self-reporting depressive symptoms; 26 EU member countries. Source: Eurostat (2016) [11].

Women’s morbidity disadvantage is evident across various indicators including self-rated health, pain, obesity and especially mental health [3,9]. This is demonstrated in Figures 2 and 3 which show that ‘bad or very bad’ self-reported general health and depressive symptoms are higher amongst women than men in all 28 European Union countries [10,11]. Women are also more likely to live with limiting long-term conditions (e.g. living with cancer or cardiovascular disease). For example, whilst men have higher cardiovascular disease mortality rates, due to women’s higher life expectancy, the average patient receiving treatment for cardiovascular disease is female and while cardiovascular disease rates are declining on average, the decline has slowed or even stalled amongst women [12]. Women’s morbidity disadvantage is particularly evident in the countries of Central and Eastern Europe, where they have higher rates of cardiovascular diseases and cancers than women in the rest of Europe, and where HIV/AIDS is also an important health risk for younger women [13]. As with men’s mortality disadvantage, there are also larger gender differences in morbidity between men and women in the lowest socio-economic groups [9].

Explanations for the gender health paradox are multiple but it is thought that both sex (biological factors) and gender (social factors) play important – and interacting – roles [14]. The literature has four broad explanations: biological, social, economic and public policy.

Biological explanations explore how some of the gender health paradox may be a result of differences between men and women in terms of their biological and genetic make-up, leading to more or less susceptibility to certain health outcomes. Firstly, it has been suggested that there are immune system differences between men and women, differential responses to oxidative stress and differences in mitochondrial fitness [15]. These may contribute to the gender health paradox – however, the biomedical evidence examining this is underdeveloped and controversial [15]. A clear example of biological pathways though is how increased morbidity for women from diseases of the connective tissue such as osteoporosis is related to reduced levels of oestrogen associated with the menopause [16]. Indeed, hormone levels have a strong impact on healthy ageing in both men and women [15]. Similarly, studies have suggested that the higher levels of depression amongst women may be partly genetic (in combination with social factors) [17].

Social explanations of the gender health paradox focus on variations in the behaviour of men and women, including those linked to constructions of masculinity and work–family roles [3]. Most directly, traditional conceptions of masculinity have meant that men are more likely to engage in health damaging risk-taking behaviours such as excessive alcohol consumption [18]. Men are also less likely to access healthcare services and more likely to present late with symptoms [19]. These behavioural differences may contribute to men’s higher mortality rates. Further, stress theories of health suggest that historically, men have been more exposed to negative health effects from workplace hierarchies, unemployment and the need to be the main breadwinner – all factors associated with an increased risk of mortality from key diseases such as cardiovascular disease [20]. Women in comparison are more likely to experience physical and mental health problems as a result of work-family strain from the dual burden of employment and caring [21]. Dual roles have been posited as health damaging with various studies finding associations between work–family conflict and physical ill health, depression, hypertension and alcohol misuse [21].

Economic explanations focus on how women are particularly hit by unfavourable socio-economic factors such as higher rates of poverty, historically lower rates of education, discrimination in the labour market and lower employment rates [22]. For example, women are more likely to be single parents, to work part-time or be unemployed (partly due to family responsibilities). They are also more likely to be precariously employed, or to be employed in low-wage parts of the economy. Subsequently women are more likely to experience poverty [23]. There is a gender pay gap in Europe – with women earning *c.* 20% less than men on average – and labour markets are still highly segregated, with women taking jobs connected to their traditional roles as caregivers or jobs with limited opportunities for advancement [24]. Research shows that these economic disadvantages are associated with higher rates of morbidity amongst women including chronic diseases and self-reported poor health whilst increased participation in the workforce can have beneficial health effects for women [25,26].

Public policy explanations focus on how public policies act as macro-level determinants of gender inequalities which shape the other social and economic factors, in turn influencing gender inequalities in health and well-being [27]. European countries have been leaders in family policy enacting various social investments focused on: childcare, parental leave, active labour market programmes and long-term care policies. These are in part implemented to reduce the gendered burden of family care-work and strengthen gender equity [28]. However, research suggests that the impacts of these social investment policies on gender inequalities in health are mixed [29,30]. In terms of mortality, public childcare provision is associated with lower cardiovascular disease mortality rates for both men and women equally, whilst government spending on paid parental leave and employment training decreases cardiovascular disease mortality for women [29]. In terms of morbidity, women’s health benefits more than men’s, from social investment policies with government investments in childcare, active labour market programmes and long-term care reducing disability levels. Publicly funded childcare also benefits men’s morbidity [30].

Of course, these explanations are not mutually exclusive of one another and nor will they be consistent across different health and wellbeing outcomes: different underpinning pathways will be responsible for gendered patterns of morbidity and mortality in different health outcomes. So for example, higher rates of suicide amongst men might be as a result of behavioural (e.g. alcohol consumption), social and economic pathways (such as the pressure to fulfil the role of breadwinner, lack of social support outside the workplace), potentially mitigated by public policy measures such as active labour market policies. Whilst women’s higher morbidity from mental health might be as a result of social (e.g. dual roles) or economic (e.g. gender pay gap) factors, again potentially mitigated by public policies such as public childcare.

## Gender inequalities in COVID-19 mortality and morbidity

The previous section provided an overview of the ‘gender health paradox’ and the competing explanations for it. In this section, the emerging data on gender-based inequalities in the COVID-19 pandemic are explored. From 1 January to 1 June 2020, there have been 1.4 million confirmed cases and 165,000 confirmed deaths from the COVID-19 pandemic in Europe [31]. The countries reporting the highest number of COVID-19 deaths are the UK, Italy, France, Spain and Belgium. Countries with the highest mortality rates are Belgium (82.9 per 100,000), Spain (58.1 per 100,000), the UK (57.9 per 100,000), Italy (55.3 per 100,000) and Sweden (43.2 per 100,000) [31]. However, there are already indications that the impact of COVID-19 in terms of infections, symptom severity and mortality is gendered – potentially reflecting the ‘gender health paradox’.

In terms of COVID-19 morbidity in Europe, women appear to be slightly more likely to be diagnosed with COVID-19. For example, up to 1 June 2020, in Germany, women accounted for 52% of confirmed cases and men 48% [32]. This may in part be due to the fact that women are disproportionately represented in the healthcare workforce whose exposure to the SARS-CoV-2 virus is high [33]. Women hold on average 90% of the jobs in the long-term care sector (LTC) [33]. A high proportion of LTC facilities across Europe and globally have reported COVID-19 outbreaks, with high rates of morbidity and case fatality in residents and high rates of staff absenteeism [34]. Indeed, confirmed cases among healthcare workers show that women are being infected in higher numbers than men: in Italy 68% of infected are women, in the USA 73%, Spain 75%, Germany 72% [35].

However, data from France and the UK shows that men were more likely to be admitted to intensive care with COVID-19 – reflecting more severe illness and disease complications [36]. In the UK, men made up 46% of diagnosed cases but almost 60% of deaths and 70% of admissions to intensive care units [37]. There are also early signs that the mortality rate from COVID-19 in Europe and elsewhere may be higher amongst men [32]. Early evidence from China, for example, suggested that the death rate was 2.8% amongst men and 1.7% amongst women [38]. This higher probability of dying from COVID-19 has also been evident in Europe and other countries such as the USA [32,37,39]. For example, the 8252 deaths in Germany were split 4572 for men and 3680 for women [32]. In the UK, deaths from COVID-19 amongst men were 50.6 per 100,000 compared to 25.5 per 100,000 for women [37]. The UK data also suggests that these gender inequalities in mortality are consistent across different socio-economic and ethnic groups [37].

To date, the explanations for gender inequalities in COVID-19 mortality has focused on social and biological factors. Early analysis in China focused on whether gender inequalities in smoking rates were the main factor behind these different death rates – as smoking is very high amongst older Chinese men and very low amongst older Chinese women – reflecting their stage in the global smoking epidemic [38]. Smoking increases the risks of respiratory diseases and complications [37, 40]. However, the European data suggests that it is wider than just smoking and now men’s higher mortality is considered to be due to their higher rates of the key clinical risk factors for COVID-19: European men have higher rates of cardiovascular disease, diabetes, chronic respiratory disease, hypertension and cancer [41]. They could also be driven by different risks of acquiring the infection – for example due to different occupational exposures such as working in heavy industry [37]. There has also been speculation that the differential death rates may be as a result of immunity or hormonal differences between men and women [37,42]. However, it should also be noted though that the relative increase in excess mortality amongst men and women in spring 2020 is similar – suggesting that whilst more men might be dying in terms of absolute numbers of deaths, the relative increase in mortality is the same for women [43]. So, caution needs to be applied to these early estimates and to speculation of biological pathways [43].

## Gender, lockdowns and the COVID-19 economic fallout

The previous section provided an overview of the emerging data on gender-based inequalities in the COVID-19 pandemic which suggests that men are disproportionately experiencing morbidity and mortality from COVID-19. This section explores the longer term consequences for gender-based health inequalities of COVID-19, as the impact of the pandemic will not just be in terms of virus-related morbidity and mortality, but also in terms of the health consequences of the actions undertaken by governments in response. Most European countries (with Sweden being a notable exception) implemented mass quarantine measures in spring 2020: so-called lockdowns. These state-imposed emergency restrictions have been of varying levels of severity but all have in common a significant increase in social isolation and confinement within the home and immediate neighbourhood and were initially implemented for 8 to 12 weeks [44]. The lockdowns and the continued social distancing measures in place have also led to an emerging economic crisis. This section provides an overview of the evidence to date on the gendered experience of the lockdown and its implications for health. It then draws on past research into recessions and health inequalities to examine the potential gendered impact that the COVID-19 economic crisis might have on future morbidity and mortality amongst men and women in Europe.

There are concerns within the public health and medical community that the lockdowns in Europe have led to higher rates of intimate partner violence, mental ill heath, and reduced healthcare access, particularly impacting on women. Within the first few weeks of lockdown, charities across Europe soon started to report increased cases of intimate partner violence, with higher rates of calls to their helplines and website visits [45]. For example, the Catalan regional government in Spain reported that calls to its helpline had risen by 20% in the first few days of the confinement period and in Cyprus, calls to a similar hotline rose 30% in the first week [45]. More alarmingly, reports have suggested that deaths from intimate partner violence doubled in the UK during the first month of lockdown: usually there are 5 to 6 deaths per month of women and children, but during the first four weeks of lockdown this increased to 16 [46].

The lockdown has proved particularly challenging for mental health, with concerns expressed by medical professionals from across Europe about the impact of extended isolation and lack of social contact [47]. This is exacerbated by rising financial insecurity and poverty – which is likely to be disproportionately impacting on women given that on average they have lower incomes [24]. The mental health impacts are also likely to be stronger for women as school closures have led to increased childcare pressures. This is particularly challenging for people who already have mental ill-health and given that women are more likely to suffer from anxiety and depression; it is possible that women’s psychological well-being has suffered disproportionately as a result of the lockdown [47]. Further, as a result of health services having to focus on combating the pandemic, there has also been a significant reduction in healthcare access for people with existing chronic conditions such as cancer or cardiovascular disease [48]. This will disproportionately impact on women as they are more likely to be living with such diseases. Similarly, access to preventative care such as breast cancer and cervical cancer screening has also been restricted in many European countries as a result of healthcare-system pressures and the need for social distancing [48].

Past research suggests that the longer-term economic fallout from the pandemic may also be gendered – with increased morbidity in women and increase mortality amongst men. The European and world economy has been severely impacted by COVID-19 – with major reductions in GDP, oil price falls and record levels of unemployment – many countries including France and Germany are already in recession [49]. This is despite the unprecedented interventionist measures undertaken by many European governments and central banks [50]. Economists fear that the economic impact will be far greater than the global financial crisis (GFC) of 2007/8 and they say that it is likely to as bad as – or worse than – the Great Depression of the 1930s [49]. The impacts of the COVID-19 economic fallout will likely be gendered, as the sectors of the economy most hard-hit include retail, tourism and restaurants, all of which disproportionately employ women [51]. Certainly, in the 2007/8 GFC, women tended to be more affected than men in those countries that experienced a severe economic recession [52].

There will also be huge health consequences from this economic crisis – which will impact men and women differently. For example, previous research into recessions – including the GFC – has found that recessions lead to increases in physical and psychological morbidity and mortality, particularly from suicide [53]. One pathway behind this is that unemployment increases during recessions and it is strongly associated with greater morbidity and mortality, particularly mental health problems (such as depression and anxiety, suicide and parasuicide), an increase in smoking and excessive alcohol consumption, and higher rates of limiting long-term illness (including cardiovascular disease) [54]. Recessions are also characterised by an increase in job insecurity and ‘precarious’ employment which is associated with stress, fatigue, backache and muscular pain, psychiatric morbidity, adverse health-related behaviours and mortality [53]. Studies suggest that the health effects of recessions vary by gender though with adverse mortality effects on men and adverse morbidity impacts on women [20]. For example, studies have found that the 1990s recession increased all-cause mortality in Swedish men but not Swedish women whilst in Japan and England, women – especially the lowest educated – suffered worse self-reported health than men during the 1990s recession and the GFC [3,55]. Suicide rates also increase substantially during recessions, particularly amongst men. During the immediate aftermath of the GFC, for example, there were over 800 additional suicides among men than would have been expected based on historical trends, and over 150 amongst women in England [56].

However, the effects of recessions on health vary by public policy response with countries such as the UK, Greece, Italy and Spain who imposed austerity (significant cuts in health and social protection budgets) after the GFC experiencing worse population health effects than those countries such as Germany, Iceland and Sweden who opted to maintain public spending and social safety nets [57]. By way of example, greater spending on social welfare has been shown to considerably reduce suicide rates during periods of economic downturn [58]. The gender and health effects of the COVID-19 recession may well therefore be experienced quite differently by men and women across Europe due to national policy variation, with more generous welfare systems protecting the health of the population and especially the most vulnerable [3]. Population health (including self-reported health, life expectancy and infant mortality rates) is enhanced in more generous welfare states and in periods of welfare state expansion such as the 1960s ‘War on Poverty’ in the USA or the period of Labour government in England in the 2000s [58–60]. In contrast, periods of welfare state contractions – such as the austerity policies pursued over the last decade in some parts of Europe – can damage population health particularly mental health and suicide [57]. This is especially the case for the most vulnerable in society – such as ethnic minority and migrant women or women and children on low incomes [61]. Currently, many European countries are providing temporary enhancements to social security (e.g. the universal basic income introduced in Denmark) or providing direct wage support (e.g. the UK’s ‘furlough’ system which provides up to 80% of wages to those temporarily laid off work) [44]. How long these enhancements last – and their gendered distribution – will have important implications for how the pandemic impacts on gender-based health inequalities in the longer term.

## Conclusion

This article has highlighted some of the emerging gender-related issues in the COVID-19 pandemic in Europe within the context of the wider international literature on the ‘gender health paradox’. It has examined the pandemic in light of the biological, social, economic and public policy pathways that shape gender-based inequalities in health. It has highlighted that although the mortality rate from COVID-19 may be higher amongst men, potentially as a result of both biological and social factors; the lockdown policies have led to higher rates of intimate partner violence, mental ill health and reduced healthcare access particularly impacting on women. Further, the longer-term economic fallout from the pandemic will also likely be gendered in terms of impacts on mortality and morbidity. So, whilst it is still too early to say with certainty (and there needs to be caution applied to the emerging estimates of COVID-19 mortality and morbidity), it does appear likely that the pandemic will exacerbate existing gender inequalities in health, acting intersectionally alongside ethnicity and socio-economic status [62]. Gender inequalities in health and the pathways underpinning them requires further empirical analysis – especially in light of the gendered patterning of COVID-19. Gender inequalities in COVID-19 (and differences in this between countries and regions) will also reflect the differences in public policy, economic conditions and social determinants. Welfare and health systems determine what services are provided to whom, and how they are financed – limited public health services, for example, aggravate health inequalities. The design of social policies and their effectiveness depends largely on other institutional arrangements, including economic policy, especially labour market regulation and fiscal policy, and the social determinants of health (e.g. education, the workplace and income). European countries were in different situations going into lockdowns during the pandemic and will start from different points in post-COVID recovery. While there is no panacea on offer, gender mainstreaming in health, economic and employment policies in general and in relation to COVID-19 crisis is recommendable [63]. Government responses can mitigate the longer-term health inequalities impacts of the pandemic through improving public health and healthcare services, increasing access to public services to support those with social or health needs, and maintaining and enhancing social security safety nets [64].
